# Prognostic value of diametrically polarized tumor-associated macrophages in multiple myeloma

**DOI:** 10.18632/oncotarget.22340

**Published:** 2017-11-09

**Authors:** Xinyi Chen, Jin Chen, Wenyan Zhang, Ruixue Sun, Ting Liu, Yuhuan Zheng, Yu Wu

**Affiliations:** ^1^ Department of Hematology, Hematology Research Laboratory, West China Hospital, Sichuan University, Chengdu, China; ^2^ Department of Rheumatology and Immunology, West China Hospital, Sichuan University, Chengdu, China; ^3^ Department of Pathology, West China Hospital, Sichuan University, Chengdu, China

**Keywords:** multiple myeloma, tumor-associated macrophages, overall response, prognosis, nomogram

## Abstract

Tumor-associated macrophages (TAMs) are correlated with the prognosis of different types of solid tumors and lymphoma, according to many clinical studies. *In vitro* experiments have demonstrated the roles of these cells in myeloma cell survival, angiogenesis, immunomodulation, drug resistance, and the interaction between malignant myeloma cells and the microenvironment. Here, we investigated the prognostic significance of TAMs in patients with multiple myeloma (MM). We evaluated the polarized functional status of bone marrow infiltrated by TAMs by immunohistochemical staining of CD68, iNOS, and CD163 in 240 patients with MM from January 2009 to December 2014. The overall response rates to chemotherapy were lower in patients with high CD68^+^ or CD163^+^ TAM densities than in those with low densities. Kaplan-Meier analysis showed that the progression-free survival (PFS, *p* = 0.001) and overall survival (OS, *p* < 0.001) of patients with low CD163^+^ TAM density were significantly higher than those of patients with high CD163^+^ TAM density. Furthermore, combined analysis of iNOS^+^ and CD163^+^ TAMs (iNOS/CD163 signature) exhibited greater power in predicting patient outcomes for both PFS (*p* < 0.001) and OS (*p* < 0.001). Moreover, Cox regression analysis identified iNOS^+^ and CD163^+^ TAMs as independent prognostic factors (*p* = 0.007, *p* < 0.001, respectively). These factors could be combined with the international staging system (ISS) to generate a predictive nomogram for patient outcomes. Our findings suggest that the mosaic of diametrically polarized TAMs is a novel independent prognostic factor that could be integrated into the evaluation of and therapy for MM.

## INTRODUCTION

Multiple myeloma (MM) is a malignant B-cell tumor characterized by the infiltration and proliferation of monoclonal plasma cells into the bone marrow. This infiltration can result in excess immunoglobulin secretion, osteolytic lesions, impaired renal function, and myelosuppression [1]. Although novel therapies have resulted in breakthrough improvements in the last decade, MM generally remains incurable. Considerable heterogeneity still exists with regard to survival, relapse, and resistance [2]. Patient factors and tumor variables could influence the prognosis of myeloma. The International Staging System (ISS), the most widely used prognostic system for MM, stratifies patients into three groups based on serum albumin and β2-microglobulin levels [3], which is well validated and easily applied; however, serum albumin and β2-microglobulin levels may not provide complete prognostic information because they do not incorporate tumor-microenvironment information.

Recently, several studies have reported that macrophages are a major cellular component of the tumor microenvironment in the pathogenesis of myeloma [4–6]. Furthermore, accumulating evidence suggests that these tumor-associated macrophages (TAMs) actively promote tumor initiation, growth, and progression [5, 7–9]. In humans, CD68, CD163, CD204 and CD206 are the major markers of macrophage lineage [10]. Infiltrating macrophages respond to the products of malignant and stromal cells and adapt to a range of activation states that can be classified within the M1/M2 polarization model [11, 12]. M1-type macrophages, which are classically activated by toll-like receptor (TLR) ligands and interferon-γ (IFN-γ), play roles in antitumor immunity. The M1 macrophage has been shown to up-regulate the expression of the IL-12, TNF-α, and inducible nitric oxide synthase (iNOS) [13]. However, M2-type macrophages, stimulated by interleukin-4 (IL-4) or IL-13, have the ability to promote tumor growth and progression. The M2 macrophages reduce the expression of iNOS and have enhanced expression of CD163 (hemoglobin scavenger receptor), CD204 (class A macrophage scavenger receptor) and CD206 (mannose receptor C type 1) [14–16]. In this study, the TAM phenotype was determined using CD68 (macrophage marker), iNOS (M1) and CD163 (M2) antibodies, respectively.

Therefore, it is plausible that knowledge of the complexity of the macrophage phenotype in the MM tumor microenvironment could help to predict prognoses in MM cases. Hence, the aim of this study was to evaluate the M1 vs. M2 phenotypes of TAMs in the early therapeutic response to chemotherapy and determine whether the TAM phenotype is correlated with survival in MM.

## RESULTS

### Correlations between TAM polarization status and clinicopathological features

Patient characteristics and correlations among immunohistochemistry (IHC) variables and clinicopathological features are summarized in Table 1. The median subject age was 63 years (range, 34–87 years) with 132 males and 108 females. Twenty-nine (12.1 %) patients were in stage IA; 36 (15 %) patients were in stage IIA; 16 (6.7 %) patients were in stage IIB; 95 (39.6 %) patients were in stage IIIA; and 64 (26.7 %) patients were in stage IIIB at diagnosis according to the Durie–Salmon (DS) staging system. Thirty-five (14.6 %) patients were at stage I, 45 (18.8 %) patients were at stage II, and 160 (66.7 %) patients were at stage III at diagnosis according to International Staging System (ISS). Thirty patients (12.5 %) received bortezomib-based or lenalidomide-based therapy, 161 patients (67.1 %) with thalidomide-based regimens, 37 patients (15.4 %) with VAD (vincristine, adriamycin and dexamethasone) and 12 patients (5.0 %) with MP (melphalan and prednisone) regimen. TAMs can be detected in all stages of MM. Although CD163^+^ TAM density was positively correlated with age (*p* = 0.028), no significant association between IHC variables and other clinicopathological factors including ISS stage, DS stage, bone destruction, renal failure and induction treatment were observed (Table 1).

**Table 1:** Correlations between immunohistochemical variables and clinicopathological characteristics

Variable	Total (n = 240)	CD68^+^ TAMs			iNOS^+^ TAMs			CD163^+^ TAMs		
		Low (n = 157)	High (n = 83)	*p*	Low (n = 128)	High (n = 112)	*p*	Low (n = 148)	High (n = 92)	*p*
Age										
Mean (years)^a^	63	63.0	61.1	0.171	62.6	62.0	0.620	61.2	64.1	**0.028**
Median	63	64	61		63	63		61	64	
Range	34-87	34-82	36-87		36-87	34-82		34-82	38-87	
≥ 65 yr-no (%)	99 (41.3 %)	68 (28.3 %)	27 (11.3 %)		52 (21.7 %)	46 (19.2 %)		54 (22.5 %)	44 (18.3 %)	
> 75 yr-no (%)	24 (10 %)	16 (6.7 %)	7 (2.9 %)		15 (6.3 %)	8 (3.3 %)		13 (5.4 %)	10 (4.2 %)	
Gender				0.235			0.145			0.088
Male	132 (55 %)	82 (34.2%)	50 (20.8 %)		76 (31.7 %)	56 (23.3 %)		75 (31.3 %)	57 (23.8 %)	
Female	108 (45 %)	75 (31.3%)	33 (13.8 %)		52 (21.7 %)	56 (23.3 %)		73 (30.4 %)	35 (14.6 %)	
DS staging system				0.261			0.293			0.436
IA	29 (12.1 %)	24 (10 %)	5 (2.1 %)		13 (5.4 %)	12 (6.7 %)		21 (8.8 %)	8 (3.3 %)	
IIA	36 (15 %)	21 (8.8 %)	15 (6.3 %)		18 (7.5 %)	18 (7.5 %)		23 (9.6 %)	13 (5.4 %)	
IIB	16 (6.7 %)	10 (4.2 %)	6 (2.5 %)		6 (2.5 %)	10 (4.2 %)		12 (5 %)	4 (1.7 %)	
IIIA	95 (39.6 %)	59 (24.6 %)	36 (15 %)		59 (24.6 %)	36 (15 %)		56 (23.3 %)	39 (16.3 %)	
IIIB	64 (26.7 %)	43 (17.9 %)	21 (8.7 %)		32 (13.3 %)	32 (13.3 %)		36 (15 %)	28 (11.7 %)	
ISS				0.644			0.108			0.886
I	35 (14.6 %)	25 (10.4 %)	10 (4.2 %)		15 (6.3 %)	20 (8.3 %)		22 (9.2 %)	13 (5.4 %)	
II	45 (18.8 %)	27 (11.3 %)	17 (7.1 %)		20 (8.3 %)	25 (10.4 %)		29 (12.1 %)	16 (6.7 %)	
III	160 (66.7 %)	105 (43.8 %)	56 (23.3 %)		93 (38.8 %)	67 (27.9 %)		97 (40.4 %)	63 (26.3 %)	
Creatinine (mg/dl)				0.493			0.185			0.349
≤ 2 mg/dl	152 (63.3 %)	97 (40.4 %)	55 (22.9 %)		86 (35.8 %)	66 (27.5 %)		95 (39.6 %)	57 (23.8 %)	
> 2 mg/dl	88(36.7 %)	60 (25 %)	28 (11.7 %)		42 (17.5 %)	46 (19.2 %)		53 (22.1 %)	35 (14.6 %)	
LDH				0.142			0.497			0.286
Normal	151 (62.9 %)	104 (43.3 %)	47 (19.6 %)		78 (32.5 %)	73 (30.4 %)		97 (40.4 %)	54 (22.5 %)	
High	89(37.1 %)	53 (22.1 %)	36 (15 %)		50 (20.8 %)	39 (16.3 %)		51 (21.3 %)	38 (15.8 %)	
Bone destruction				0.89			0.70			0.52
≤ 3 lesions	110 (45.8 %)	64 (26.7 %)	46 (19.2 %)		49 (20.4 %)	61 (25.4 %)		48 (20 %)	62 (25.8 %)	
> 3 lesions	130 (54.2 %)	77 (32.1 %)	53 (22.1 %)		54 (22.5 %)	76 (31.7 %)		63 (26.3 %)	67 (27.9 %)	
Induction treatment				0.943			0.142			0.298
MP	12 (5.0 %)	7 (4.5 %)	5 (6.0 %)		8 (6.3 %)	4 (3.6 %)		5 (3.4 %)	7 (7.6 %)	
VAD	37 (15.4 %)	26 (16.6 %)	11 (13.3 %)		26 (20.3 %)	11 (9.8 %)		23 (15.5 %)	14 (15.2 %)	
Thalidomide-based regimens (TD & MPT)	161 (67.1 %)	104 (66.2 %)	57 (68.7 %)		76 (59.4 %)	85 (75.9 %)		104 (70.3 %)	57 (62.0 %)	
Novel drugs (LD, VD & PAD)	30 (12.5 %)	20 (12.7 %)	10 (12.0 %)		18 (14.0 %)	12 (10.7 %)		16 (10.8 %)	14 (15.2 %)	

*TAMs* tumor-associated macrophages, *DS* Durie Salmon Staging System, *ISS* International Staging System, *LDH* lactate dehydrogenase, *MP* melphalan with prednisone; *VAD* vincristine with adriamycin plus dexamethasone, *TD* thalidomide with dexamethasone, *MPT* melphalan with prednisone plus thalidomide, *LD* lenalidomide with dexamethasone, *VD* bortezomib with dexamethasone, *PAD* bortezomib with adriamycin plus dexamethasone.

^a^ Student's t test; Chi-square test for all other analyses

### Immunohistochemical detection of macrophages

**Figure 1:**
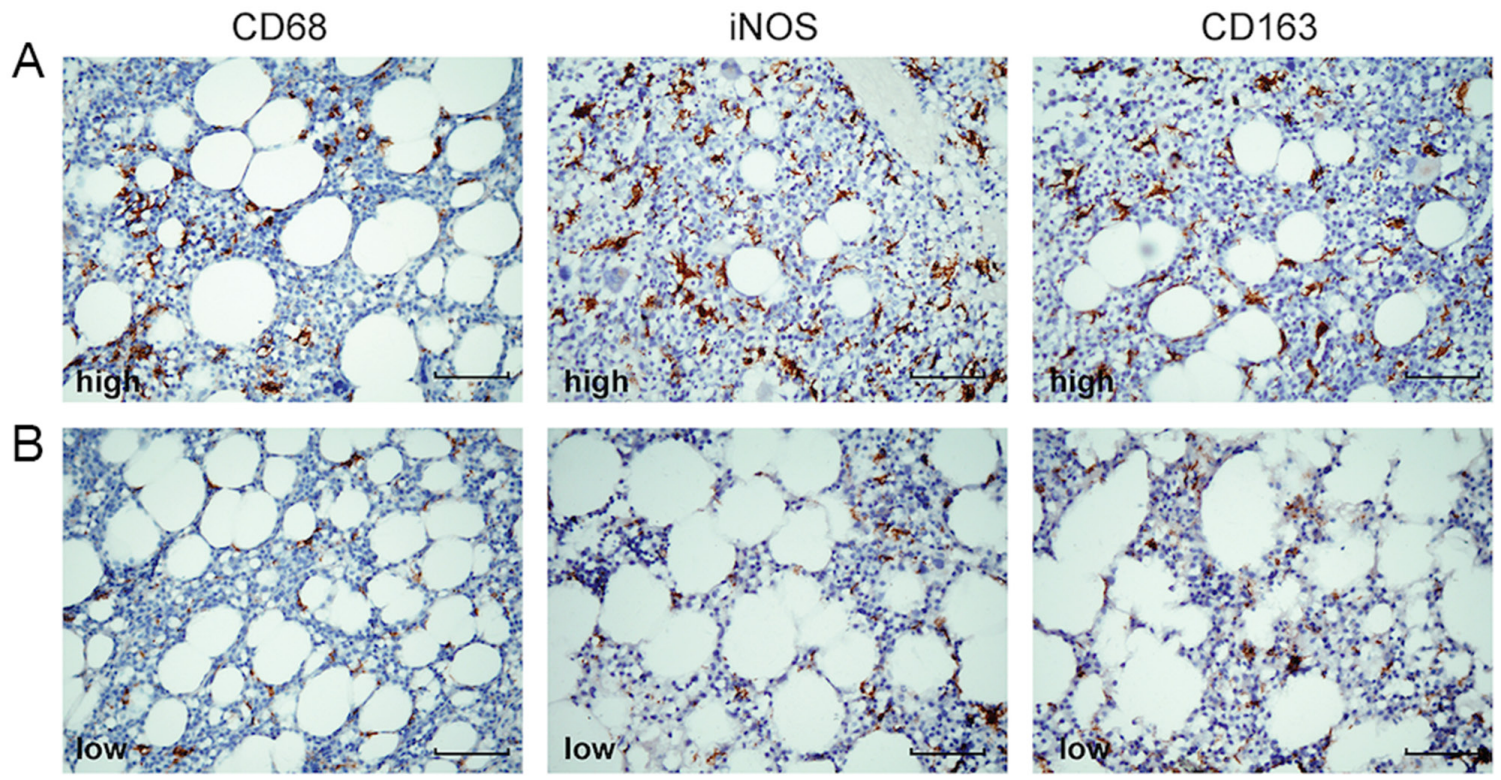
Representative immunohistochemical images of low- or high-density CD68^+^, iNOS^+^, and CD163^+^ tumor-associated macrophages in bone marrow Consecutive sections were used for immunohistochemical studies: **(A)** CD68^high^, iNOS^high^, CD163^high^; **(B)** CD68^low^, iNOS^low^, CD163^low^. Positive macrophages were stained brown (× 400 magnification). The scale bars represent 50 μm.

CD68, iNOS, and CD163 positive staining were observed in the cytoplasm of macrophages (Figure 1). Generally, CD68^+^ cells were more abundant than iNOS^+^ or CD163^+^ cells. In order to assess whether the markers chosen actually detect different cellular subsets of macrophages, both paraffin-embedded slides and bone marrow smears from patients with MM were double stained for iNOS or CD163 with CD68. The immunofluorescent images of confocal microscopy showed co-expression of iNOS or CD163 with CD68 on the same cell on both paraffin-embedded slides (Supplementary Figure 1) and bone marrow smears (Supplementary Figure 2).

### Correlations between TAM polarization and clinical responses

The overall response to chemotherapy was lower in patients with high CD68^+^ or CD163^+^ TAM densities than in patients with low densities (Table 2). Compared to patients with low CD68^+^ TAM densities, patients with high CD68^+^ TAM densities had significantly decreased frequencies of overall response (complete, very good partial response, or partial response; 26.5 % vs. 68.8 %, *p* < 0.001). Similarly, patients with high CD163^+^ TAM density had a lower overall response rate than those with low CD163^+^ TAM density (23.9 % vs. 73.0 %, *p* < 0.001). In contrast, higher numbers of iNOS^+^ TAMs were correlated with higher overall response rates (69.6 % vs. 40.6 %, *p* < 0.001). Moreover, the median number of iNOS^+^ TAMs increased statistically and a decline of CD163^+^ TAMs was observed in those patients who achieved overall responses after induction treatment (Supplementary Figure 3). We also analyzed the proportion of different treatment regimens in patients with overall responses, stable diseases and progressive diseases respectively. No statistical difference was found in these three groups in terms of treatment regimens (Supplementary Figure 4).

**Table 2:** Correlations between immunohistochemical variables and response rates after induction therapy

Variables	CD68^+^ TAMs		iNOS^+^ TAMs		CD163^+^ TAMs	
	Low (157)	High (83)	Low (128)	High (112)	Low (148)	High (92)
Overall response	108 (68.8 %)^*^	22 (26.5 %)^*^	52 (40.6 %)^*^	78 (69.6 %)^*^	108 (73.0 %)^*^	22 (23.9 %)^*^
Complete response	21 (13.4 %)	3 (3.6 %)	11 (8.6 %)	13 (10.7 %)	22 (14.9 %)	2 (2.2 %)
Very good partial response	2 (1.3 %)	2 (2.4 %)	3 (2.3 %)	1 (0.9 %)	3 (2.0 %)	1 (1.1 %)
Partial response	85 (54.1 %)	17 (20.5 %)	38 (29.7 %)	64 (57.1 %)	93 (62.8 %)	19 (20.7 %)
Stable disease	15 (9.6 %)	12 (14.5 %)	15 (13.4 %)	12 (10.7 %)	13 (8.8 %)	14 (15.2 %)
Progressive disease	33 (21.0 %)	46 (55.4 %)	58 (45.3 %)	21 (18.8 %)	24 (16.2 %)	55 (59.8 %)
Relapse	1 (0.6 %)	3 (3.6 %)	3 (2.3 %)	1 (0.9 %)	3 (2.0 %)	1 (1.1 %)

^*^ *p* < 0.001 by Pearson's χ^2^ test for the comparison between each two groups.

To investigate the effect of M1/M2 macrophage phenotype on the clinical response, patients were classified into four groups according to their iNOS and CD163 TAM densities: group I, high iNOS^+^ but low CD163^+^ TAM densities; group II, both low densities; group III, both at high densities; group IV, low iNOS^+^ but high CD163^+^ TAM densities. A significant difference was observed in the rate of overall response among the four groups (*p* < 0.001). The overall response rates for group I, II, III, and IV were 80.8 %, 64.3 %, 44.1 %, and 12.1 %, respectively (Table 3).

**Table 3:** Correlations between iNOS/CD163 signature and response rates after induction therapy

Variables	iNOS/CD163 signature			
	I (78)	II (70)	III (34)	IV (58)
Overall response — no. (%)	63 (80.8 %)^*^	45 (64.3%)^*^	15 (44.1 %)^*^	7 (12.1 %)^*^
Complete response	7 (9.0 %)	6 (8.6 %)	5 (14.7 %)	6 (10.3 %)
Very good partial response	1 (1.3%)	2 (2.9 %)	0	1 (1.7 %)
Partial response	55 (70.5 %)	37 (52.9 %)	10 (29.4 %)	0
Stable disease	7 (9.0 %)	6 (8.6 %)	5 (14.7 %)	9 (15.3 %)
Progressive disease	7 (9.0 %)	17 (24.3 %)	14 (41.2%)	41 (69.5 %)
Relapse	1 (1.3 %)	2 (2.9 %)	0	1 (1.7 %)

Patients were classified into four groups according to their iNOS^+^ and CD163^+^ TAM densities: group I, high iNOS^+^ but low CD163^+^ TAM densities; group II, both low densities; group III, both high densities; group IV, low iNOS^+^ but high CD163^+^ TAM densities. ^*^ *p* < 0.001 by Pearson's χ^2^ test for the comparison between these four groups.

### Prognostic value of M1 vs, M2 TAM phenotypes

**Figure 2:**
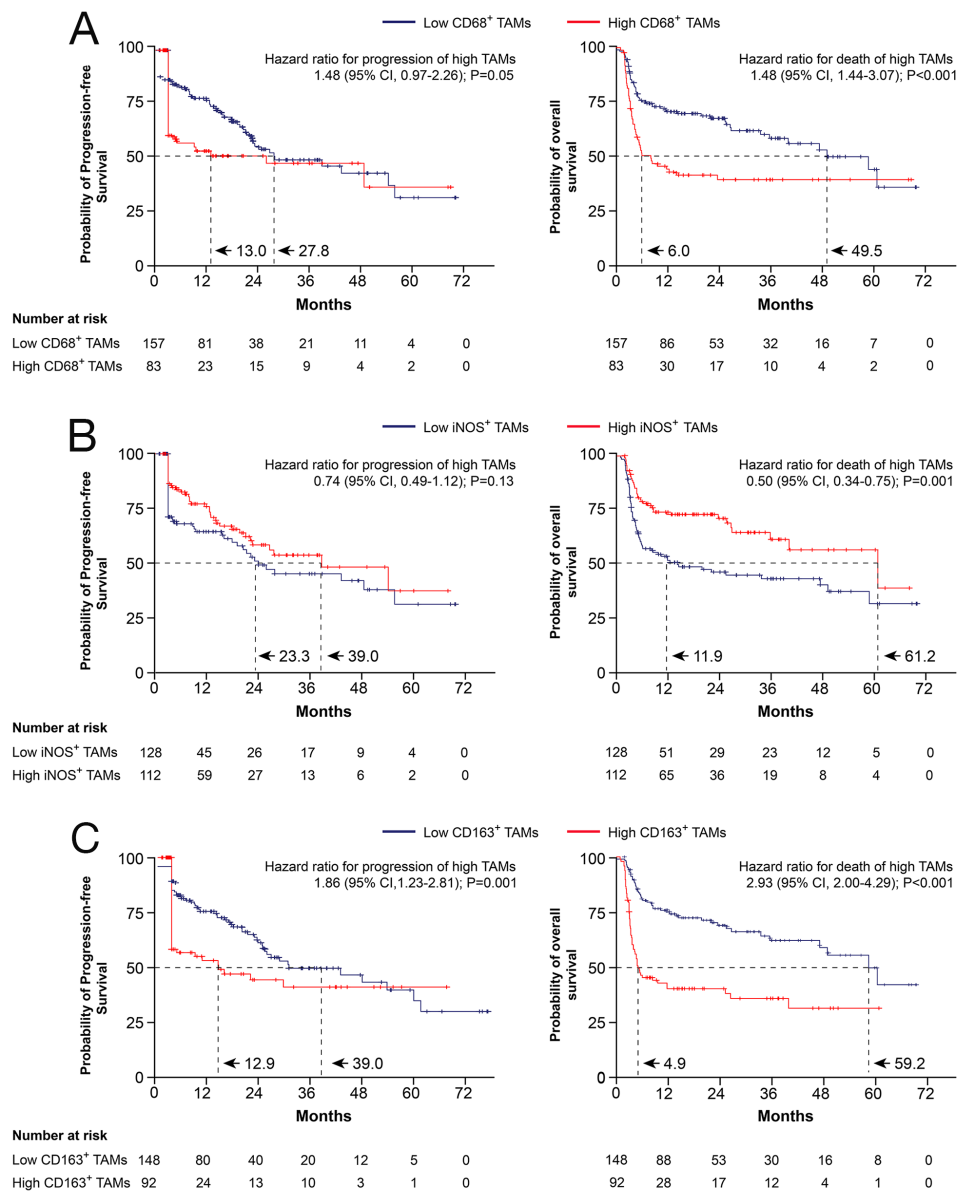
Kaplan-Meier survival curves showing the PFS and OS probabilities based on bone marrow CD68^+^, iNOS^+^, and CD163^+^ TAM densities (**A**, **B**, and **C**). The *p* values were calculated using the log-rank test. TAMs: tumor-associated macrophages; 95 % CI: 95 % confidence interval.

Kaplan-Meier analysis indicated that high CD163^+^ TAM density was associated with reduced PFS (Figure 2C; *p* = 0.001), and that high CD68^+^ TAM density only had borderline prognostic significance (Figure 2A; *p* = 0.05), whereas high iNOS^+^ TAM density was not correlated with PFS (Figure 2B; *p* = 0.13). Similarly, Kaplan-Meier analysis indicated that high CD68^+^ or CD163^+^ TAM densities were associated with reduced OS (Figure 2A, C; *p* < 0.001 and *p* < 0.001, respectively), whereas high iNOS^+^ TAM densities in MM bone marrow biopsies were associated with increased OS (Figure 2B; *p* = 0.001).

**Figure 3:**
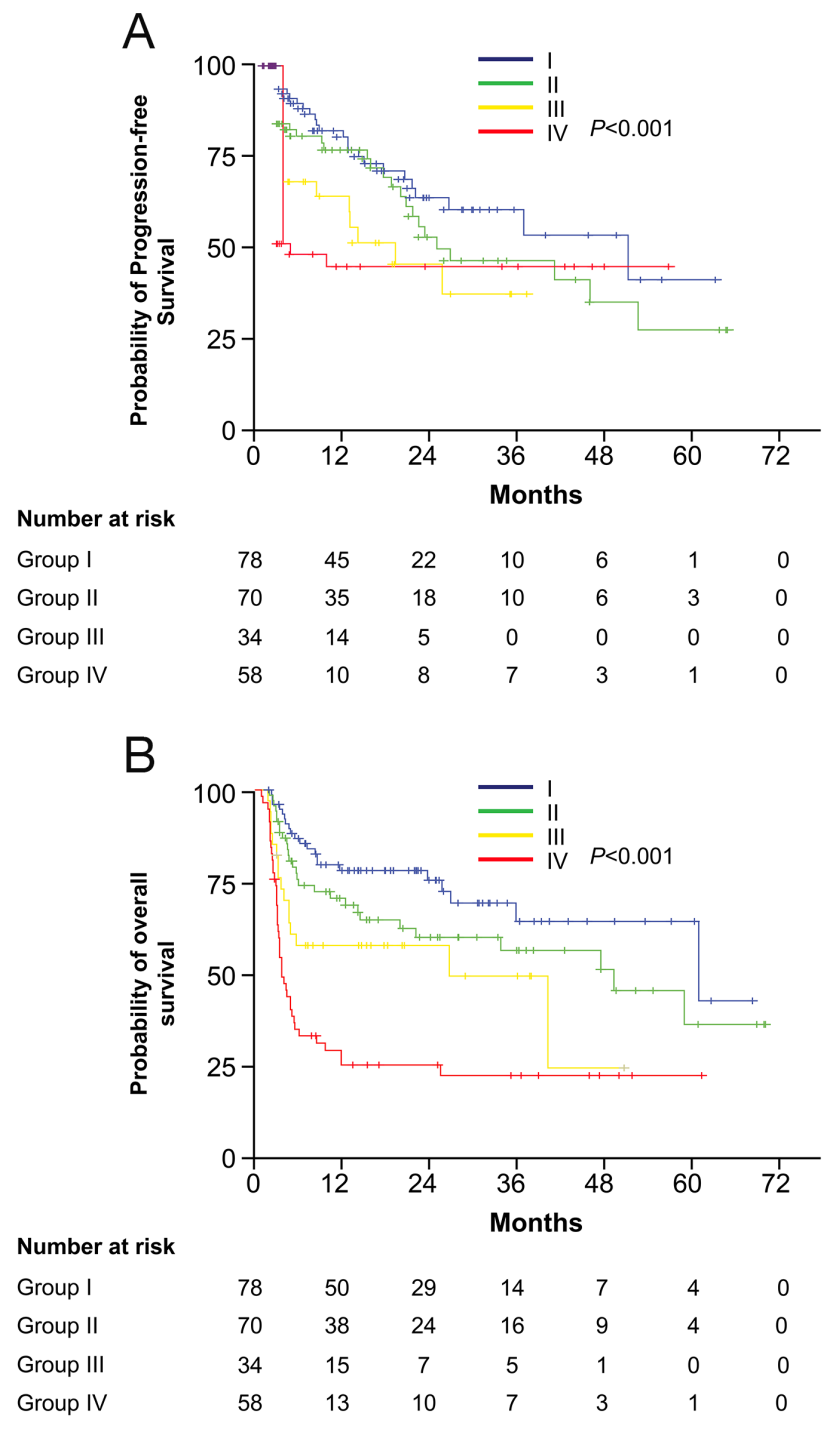
Kaplan-Meier curves showing PFS **(A)** and OS **(B)** probabilities based on the combination of iNOS^+^ and CD163^+^ TAM densities in all patients. Patients were classified into four groups according to their iNOS^+^ and CD163^+^ TAM densities: group I, high iNOS^+^ but low CD163^+^ TAM densities; group II, both low densities; group III, both high densities; group IV, low iNOS^+^ but high CD163^+^ TAM densities. The *p* values were calculated using the log-rank test.

To investigate the effect of the M1 vs. the M2 TAM phenotype on prognosis, Kaplan-Meier analysis was performed in the four TAM groups described above. Significant differences in both PFS (*p* < 0.001) and OS (*p* < 0.001) were found among the four groups (Figure 3). The median PFS values for groups I, II, III, and IV were 54.6, 26.0, 14.2, and 3.0 months, respectively. Furthermore, the median OS values for groups I, II, III, and IV were 61.2, 49.5, 26.8, and 3.7 months, respectively. Thus, an iNOS^high^/CD163^low^ TAM phenotype is consistent with an M1 TAM-predominant phenotype in the bone marrow microenvironment and may predict a favorable therapeutic outcome. In contrast, the iNOS^low^/CD163^high^ TAM profile is consistent with an M2-predominant phenotype and may predict a poorer therapeutic prognosis. Patients in groups II and III had intermediate survival rates, and the impact of iNOS^+^ TAMs on the prognosis was likely counterbalanced by CD163^+^ TAMs, and vice versa.

### Univariate and multivariate survival analysis

Table 4 lists the univariate and multivariate analyses of potential prognostic factors. The clinical factors that were significantly associated with reduced OS were ISS state (*p* = 0.002), CD68^+^ TAMs (*p* < 0.001), iNOS^+^ TAMs (*p* = 0.001), CD163^+^ TAMs (*p* < 0.001), and iNOS/CD163 signature (*p* < 0.001). Moreover, to avoid overlap between CD68^+^ TAMs and iNOS^+^ or CD163^+^ TAMs, two independent multiple analyses were performed (Table 4 and Supplementary Table 1). ISS stage (*p* = 0.001), iNOS^+^ TAMs (*p* = 0.007), CD163^+^ TAMs (*p* < 0.001), and iNOS/CD163 signature (*p* < 0.001) were identified as independent prognostic factors for OS after adjustment of covariates. Similarly, after backward stepwise variable selection, CD163^+^ TAMs (*p* = 0.005) and iNOS/CD163 signature (*p* = 0.025) were retained in the model as independent factors for PFS (Table 4).

**Table 4:** Univariate and multivariate Cox regression analyses of potential prognostic factors for MM

Variable	Progression-free survival				Overall survival			
	Univariate analysis		Multivariate analysis		Univariate analysis		Multivariate analysis	
	HR (95 % CI)	*p*	HR (95 % CI)	*p*	HR (95 % CI)	*p*	HR (95 % CI)	*p*
Gender	1.00 (0.67-1.5)	1.00	0.83 (0.48-1.45)	0.52	1.13 (0.77-1.65)	0.54	0.70 (0.40-1.22)	0.21
Age	1.01 (0.99-1.0)	0.26	1.01 (0.98-1.03)	0.52	1.01 (0.99-1.03)	0.17	1.01 (0.98-1.03)	0.67
ISS^a^		™0.12		0.11		**0.002**		**0.001**
II vs. I	1.21 (0.57-3.2)	0.63	1.32 (0.61-2.86)	0.49	0.79 (0.35-1.79)	0.57	1.01 (0.43-2.32)	0.94
III vs. I	1.76 (0.95-1.8)	0.08	1.87 (0.98-3.54)	0.06	2.05 (1.12-3.76)	0.02	2.41 (1.29-4.50)	0.006
Creatinine	1.16 (0.77-1.7)	0.48	1.12 (0.71-1.76)	0.63	1.27 (0.86-1.84)	0.224	1.13 (0.74-1.71)	0.57
LDH	0.99 (0.65-1.5)	0.97	1.19 (0.64-2.21)	0.58	1.26 (0.86-1.85)	0.241	1.62 (0.90-2.92)	0.11
iNOS^+^ TAMs (high vs. low)	0.74 (0.49-1.1)	0.16	0.78 (0.51-1.19)	0.25	0.50 (0.34-0.75)	**0.001**	0.57 (0.38-0.86)	**0.007**
CD163^+^ TAMs (high vs. low)	1.86 (1.23-2.8)	**0.003**	1.84 (1.20-2.83)	**0.005**	2.93 (2.00-4.30)	**< 0.001**	2.87 (1.93-4.26)	**< 0.001**
iNOS/CD163 signature^b^		**0.02**		**0.025**		**< 0.001**		**< 0.001**
II vs. I	1.37 (0.80-2.3)	0.25	1.31 (0.76-2.25)	0.33	1.58 (0.90-2.78)	0.11	1.47 (0.83-2.60)	0.19
III vs. I	2.03 (1.08-3.8)	0.03	1.91 (1.00-3.65)	0.05	2.35 (1.22-4.50)	0.01	2.26 (1.16-4.44)	0.017
IV vs. I	2.30 (1.31-4.0)	0.004	2.35 (1.31-4.24)	0.004	4.73 (2.80-8.00)	< 0.001	4.75 (2.76-8.16)	< 0.001

*HR* hazard ratio, *95 % CI* 95 % confidence interval, *ISS* International Staging System, *LDH* lactate dehydrogenase, *TAMs* tumor associated macrophages. ^a^ Reference group. ^b^ Patients were classified into four groups according to their iNOS and CD163 TAM densities: group I, high iNOS^+^ but low CD163^+^ TAM densities; group II, both low densities; group III, both high densities; group IV, low iNOS^+^ but high CD163^+^ TAM densities.

### Predictive nomogram model for PFS and OS of MM patients

**Figure 4:**
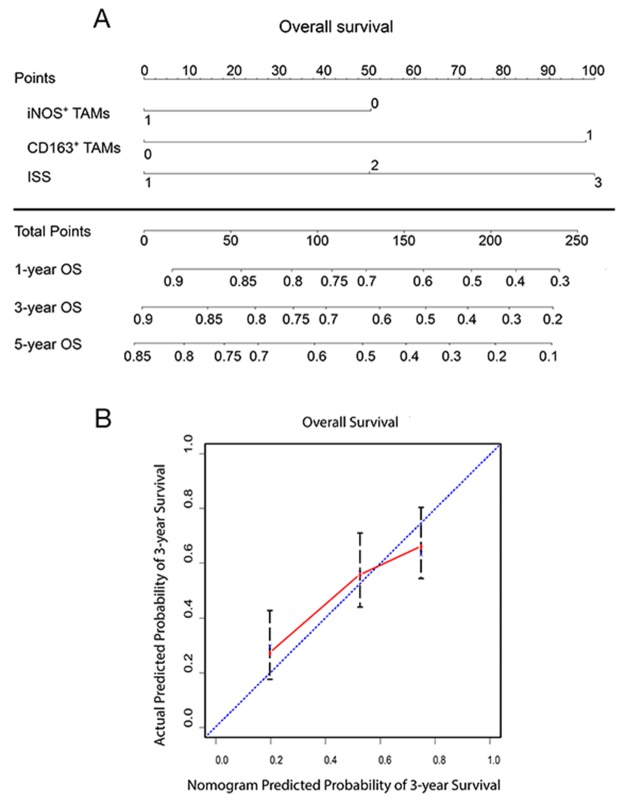
Prognostic nomogram to predict OS in patients with multiple myeloma **(A)** Nomogram generated to predict outcomes integrated with polarized TAMs (iNOS and CD163; 0 represents low density, 1 represents high density) and ISS (1 represents stage I, 2 represents stage II, 3 represents stage III). e.g. if one individual was found to have high iNOS^+^ TAMs, low CD163^+^ TAMs and ISS II at diagnosis, the nomogram score would be 150 and the 1-year OS probability would be slightly over 60%. **(B)** Calibration curve for nomogram-predicted and observed three-year outcomes.

To provide a quantitative method to better stratify patients with different prognoses, we constructed a nomogram of OS integrating all significant independent factors identified in the multivariate analysis (Figure 4A). The calibration plot for the probability of and OS at five years showed optimal agreement between predictions by the nomogram and observed outcomes (Figure 4B). The concordance-index (C-index) was 0.643 in the nomogram model, compared to 0.488 in the ISS stage model. All of these results indicated that incorporation of TAM phenotypes into the ISS could further stratify patients by prognosis.

## DISCUSSION

Accumulating evidence has indicated that activated macrophages, together with other immune cells, are central to tumor-associated inflammation, which plays important roles in the pathogenesis of many tumors [17, 18]. Interestingly, macrophages can acquire different phenotypes with partly conflicting properties; M1 macrophages play roles in host defense from a variety of bacteria and viruses and in anticancer immunity, whereas M2 macrophages tune inflammatory responses, enhance angiogenesis, and promote tumor progression [14, 19]. The functional status of TAMs in tumors is not immutable, and dynamic changes in phenotype may occur. TAMs shift functional phenotypes in response to various microenvironmental signals generated from tumor and stromal cells [20, 21]. Indeed, macrophages often express a mixed M1/M2 phenotype in response to signals in the microenvironment, and the M1 and M2 phenotypic states reflect extremes of a continuum [19, 22].

We showed that the infiltration of diametrically polarized TAMs influences the clinical response to dexamethasone-containing chemotherapy in patients with MM. To our knowledge, this study represents the largest set of clinical observations of TAMs and myeloma to date. Specifically, TAMs expressing CD68 and CD163 are negatively correlated with the response to chemotherapy, and the presence of iNOS in TAMs is positively correlated with this response. Furthermore, bone marrow infiltration by diametrically polarized TAMs influences both PFS and OS. We demonstrated that TAMs identified by CD163-positive staining have a significant negative correlation with PFS, while CD68 or iNOS have no significant correlation with PFS. TAMs expressing CD68 and CD163 are negatively correlated with OS, while iNOS is positively correlated with OS. Moreover, iNOS^+^ and CD163^+^ TAMs were shown to be independent prognostic factors by Cox regression analysis. Furthermore, we showed that identification of bone marrow macrophage phenotypes using iNOS/CD163 signatures for M1 or M2 TAMS may predict therapeutic outcomes. We incorporated TAM phenotypes into the established ISS to generate a nomogram that can more precisely quantify prognostic risk after chemotherapy for MM patients. However, more data are required before TAM phenotypes are incorporated into prognostic models.

It is believed that TAMs in the tumor microenvironment differentiate toward a protumoral, M2 phenotype [23]. In contrast, our data demonstrate that both iNOS^+^ and CD163^+^ cells were abundant in many cases, suggesting a mixed M1/M2 macrophage population in the MM bone marrow. These data indicate that the absolute cell numbers may not precisely reflect macrophage polarization in the tumor microenvironment. Thus, the iNOS/CD163 signature was used to identify M1/M2 macrophages in patient tumors. An iNOS^high^/CD163^low^ mosaic predominated by M1 TAMs was associated with a favorable prognosis (both PFS and OS). In contrast, an iNOS^low^/CD163^high^ mosaic predominated by M2 TAMs was correlated with poorer outcomes. Additionally, iNOS^high^/CD163^high^ and iNOS^low^/CD163^low^ TAMs in the same tumor were consistent with a mixed M1/M2 phenotype and were associated with intermediate survival.

Recently, clinical studies based on M2 macrophages have provided more consistent results in many different tumors [8, 9, 24, 25]. Although two studies have reported that CD68^+^ and CD163^+^ TAMs have a negative effect on OS, they do not discriminate between the M1 and M2 subsets [26, 27]. However, two studies from the US and Europe showed that CD163 measured by IHC or serum is negatively correlated with MM patient survival [27, 28]. In this study, we investigated the prognostic significance of the M1/M2 phenotype in patients with MM and found that the M1/M2 phenotype may better stratify patients and provide more prognostic information than enumeration of TAMs. As our data show that MM patients displaying the iNOS^high^/CD163^low^ phenotype had a much better prognosis than patients with the iNOS^low^/CD163^high^ phenotype, immunological intervention to tip the macrophage balance toward a tumoricidal M1 phenotype is a promising adjuvant therapy for MM. For example, IFN-γ could be administered to reverse the immunosuppressive and protumoral properties of M2 macrophages [29]. In addition, suppression of the nuclear factor-κB (NF-κB) pathway (e.g., by a proteasome inhibitor) to switch TAMs to an M1 phenotype could also be used clinically [30].

Nomograms have been accepted as reliable tools to quantify risk by incorporating and illustrating important factors for oncologic prognoses [31, 32]. In our study, we figured out the nomogram to show the impact of some clinicopathological parameters on the prognosis of myeloma patients. In our study, the prognosis of individual patient could be well predicted via combining M1/M2 macrophage phenotypes with ISS parameter together compared to using ISS alone. Moreover, these results may help clinicians better identify patients who require more aggressive therapy or more intensive follow-up. However, more prospective studies are needed to validate this nomogram significance. This study has limitations because the data on cytogenetic and fluorescence *in situ* hybridization (FISH) markers were not adequate to include in the analysis.

In conclusion, our study demonstrated that the M1/M2 ratio of TAMs is a novel independent prognostic factor for MM. The nomogram based upon this ratio could be used with the ISS to more precisely quantify prognostic risk with regard to counseling patients, stratifying patients for therapies, and customizing follow-up.

## MATERIALS AND METHODS

### Patients and specimens

Two hundred forty consecutive MM patients treated at West China Hospital of Sichuan University from January 2009 to December 2014 were enrolled in our research. Informed consent was obtained from all patients or their first-degree relatives to have their bone marrow sample evaluated. Patients with newly diagnosed multiple myeloma were eligible for this cohort study. Other inclusion criteria were sufficient clinical and pathological data and life expectancy longer than 3 months. The main exclusion criteria included a history of other cancers within the past 3 years. Patients were treated according to IMWG recommendations for global myeloma care [33]. Archived paraffin-embedded bone marrow sections were collected from the Department of Pathology, West China Hospital. Clinical characteristics were collected retrospectively by reviewing the patients’ medical records. For each patient, the following clinicopathological information was collected: age, gender, ISS classification, creatinine level, and lactate dehydrogenase (LDH) level. Patients were followed up until April 2015. The study protocol was approved by the ethical committee of West China Hospital. Progression-free survival (PFS), overall survival (OS), and the response to chemotherapy were the main endpoints in the study. PFS was calculated until the date of disease progression, death from any cause during treatment, or data censoring at the last date on which the patient was known to be free of disease progression. OS was calculated until the date of death from any cause or data censoring at the last date on which the patient was known to be alive [34]. The response to therapy was assessed after three mainly dexamethasone-containing chemotherapeutic cycles according to the International Uniform Response Criteria for Multiple Myeloma [35]. The Supplementary Table 2 and 3 showed no significant association between ISS or iNOS/CD163 signature and clinicopathological factors.

### Immunohistochemistry and evaluation

Formalin-fixed, paraffin-embedded bone marrow specimens from 240 patients were obtained from the Department of Pathology, West China Hospital, Sichuan University. The marrow paraffin blocks were cut into 4-μm sections, deparaffinized using xylene, and rehydrated with an ethanol gradient. After the endogenous peroxidase was retrieved and blocked, sections were treated with non-specific staining with 3 % (v/v) H_2_O_2_ and normal goat serum. Primary monoclonal antibodies against human CD68 (KP1, Dako, Glostrup, Denmark, 1:100), iNOS (clone KP1, Abcam, Cambridge, MA, USA; 1:50), CD138 (clone Mi15, Maxim, Fuzhou, China, 1:100) and CD163 (clone KP1, Abcam, Cambridge, MA, USA, dilution 1:100; clone 10D6, Novocastra, Japan, dilution 1:100) were applied overnight in a moist chamber at 4°C. Afterwards, the slides were incubated with a corresponding HRP-labeled secondary antibody using the Envision System reagents for 30 min at room temperature. Then, they were stained with DAB for 3-5 min in a wet chamber and counterstained with hematoxylin for 8-10 s. Later, the slides were dehydrated in alcohol and coverslipped. The immunofluorescence double staining of iNOS or CD163 and CD68 in both bone marrow paraffin-embedded sections and smears were analyzed to evaluate the co-expression pattern of the three different macrophage phenotypes under confocal microscope (Nikon A1).

Two independent pathologists without any knowledge of the clinical data assessed the IHC slides and counted cells using a computerized image system consisting of an Olympus CCD camera connected to an inverted microscope (Nikon, Eclipse TS 100, Japan). First, the bone marrow sections from all patients were reviewed based on their consecutive H&E staining and a set panel of IHC staining including anti-CD138 antibody (supplementary Figure 5A, 5B) to diagnose myeloma and outline the plasma cell distribution pattern under 400 magnification. To count the macrophages, the IHC sections were first evaluated at low power (× 100), and five representative plasma cell-rich areas (“hot spots”) were then identified. The images were captured at the maximum resolution of 4080 × 3072 pixels at × 40 magnification in each case, and the average of the five “hot spot” measurements was used for data analysis (Supplementary Figure 6). In this study, the median count (range) of CD68, iNOS and CD163 staining were 44 (17-70), 32 (8-54) and 36 (17-57) at 400 magnification (supplementary Figure 7). The median extent of infiltration (40/field) in each histologic location was used as the cut-off point for assigning patients to low- and high-TAM-density groups [36].

### Statistical analysis

Data analysis was performed with SPSS 21.0 and R (http://www.r-project.org/). Pearson's χ^2^ test or Fisher's exact test were used to compare correlations between IHC variables and clinicopathological characteristics. Kaplan–Meier analysis with the log-rank test was applied to compare survival curves. The Cox regression model was used to perform multivariate analysis of prognostic factors. On the basis of the results of the multivariable analysis, a nomogram was formulated with R using the “rms” package. A calibration plot was generated to examine the performance of the nomogram. The model performance for predicting outcome was evaluated by calculating the concordance index (C-index). The value of the C-index ranges from 0.5 to 1.0, with 0.5 indicating a random chance and 1.0 indicating a perfect ability to correctly discriminate the outcome with the model. Calibration of the nomogram for 3-year OS was performed by comparing the predicted survival with the observed survival after bias correction [37]. A two-sided *p* value less than 0.05 was considered statistically significant.

## SUPPLEMENTARY MATERIALS FIGURES AND TABLES


