# Supplementary material for: Prognostic value of diametrically polarized tumor-associated macrophages in multiple myeloma
Source: Oncotarget. 2017 Nov 9;8(68):112685–96. doi: 10.18632/oncotarget.22340 (PMC5762541; doi:10.18632/oncotarget.22340)
Supplement: Supplementary file 1 [file oncotarget-08-112685-s001.pdf]

Prognostic value of diametrically polarized tumor-associated macrophages in multiple myeloma

SUPPLEMENTARY MATERIALS

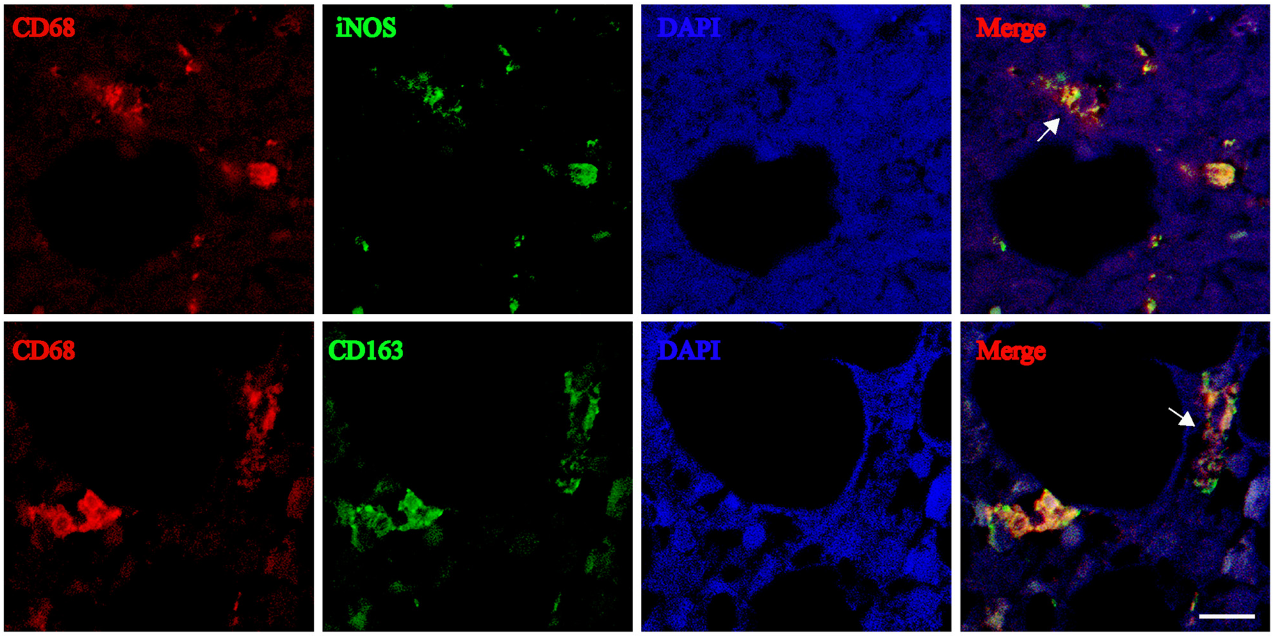

Supplementary Figure 1: Representative immunofluorescence images of M1 and M2 tumor-associated macrophages in bone marrow paraffin-embedded section analyzed by confocal microscopy. Cells stained with monoclonal anti-human CD68 antibody (red channel), monoclonal anti-human iNOS antibody (green channel) and DAPI in upper row; cells stained with monoclonal anti-human CD68 antibody (red channel), monoclonal anti-human CD163 antibody (red channel) and DAPI in lower row. White arrows point to co-expressing cells. The scale bar represents 10 µm.

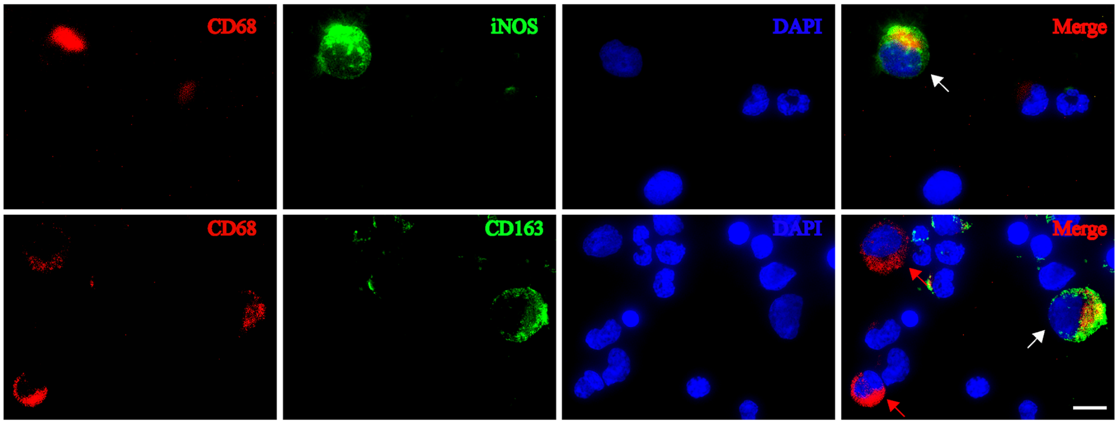

Supplementary Figure 2: Representative immunofluorescence images of M1 and M2 tumor-associated macrophages in bone marrow smear analyzed by confocal microscopy. Cells stained with monoclonal anti-human CD68 antibody (red channel), monoclonal anti-human iNOS antibody (green channel) and DAPI in upper row; cells stained with monoclonal anti-human CD68 antibody (red channel), monoclonal anti-human CD163 antibody (red channel) and DAPI in lower row. White arrows point to co-expressing cells. Red arrows point to CD68 single-positive cells. The scale bar represents 10 µm.

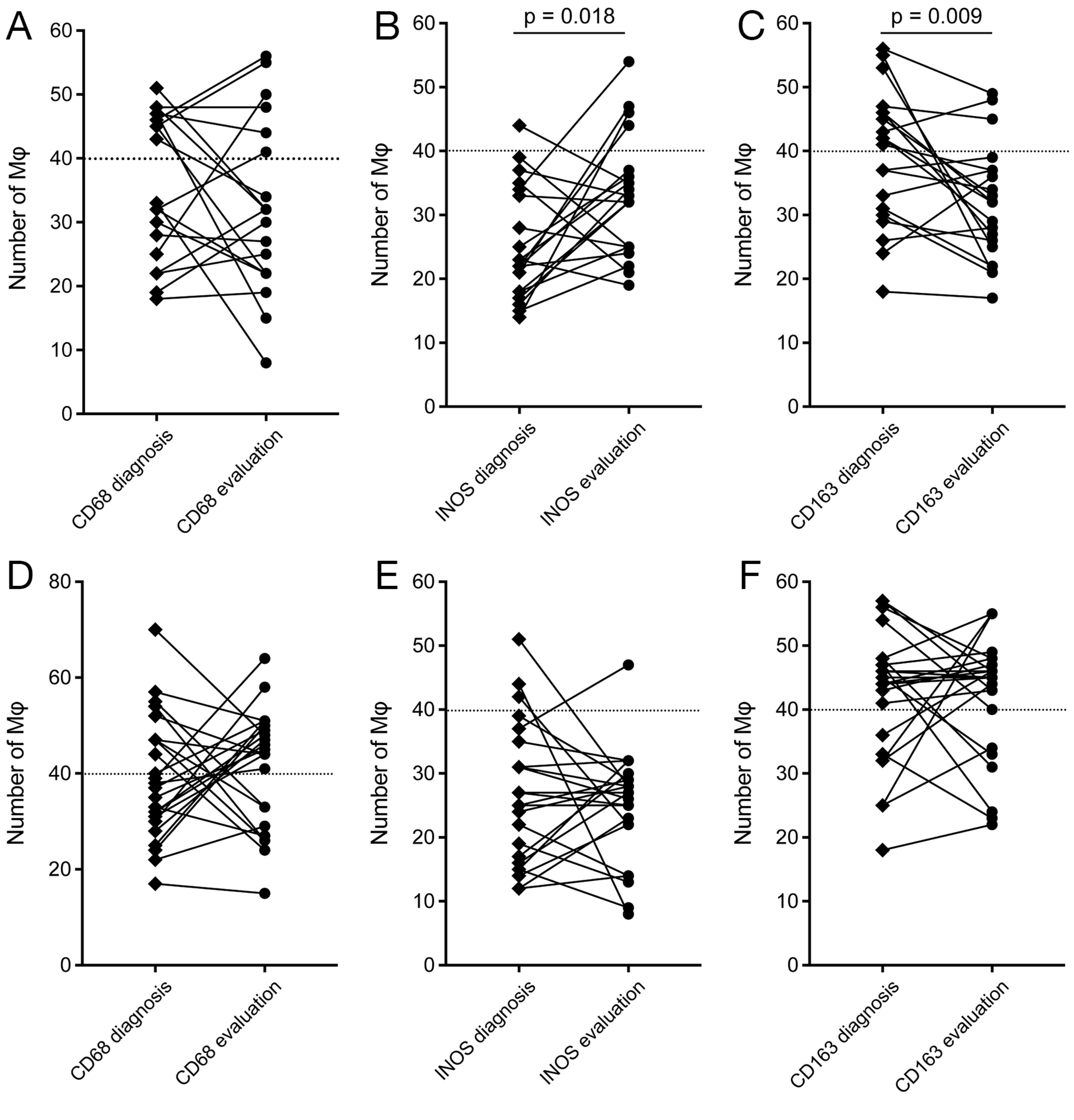

Supplementary Figure 3: The median number of TAMs at diagnosis and at evaluation after treatment. Another small cohort study enrolling forty-four patients who underwent bone marrow biopsy both at diagnosis and at evaluation after first three cycles of chemotherapy was performed. Patients were divided into two groups: responding group (20 patients) who got overall responses and non-responding groups (24 patients) who evaluated as stable diseases or progressive diseases after three cycles of chemotherapy. The upper row showed the changes of three types of macrophages in the responding group (**A** CD68+ TAM, **B** iNOS+ TAM, **C** CD163+ TAM), while the lower row indicated the changes of TAMs in the non-responding group (**D** CD68+ TAM, **E** iNOS+ TAM, **F** CD163+ TAM). $p < 0.05$ by paired t test for comparison between each two groups.

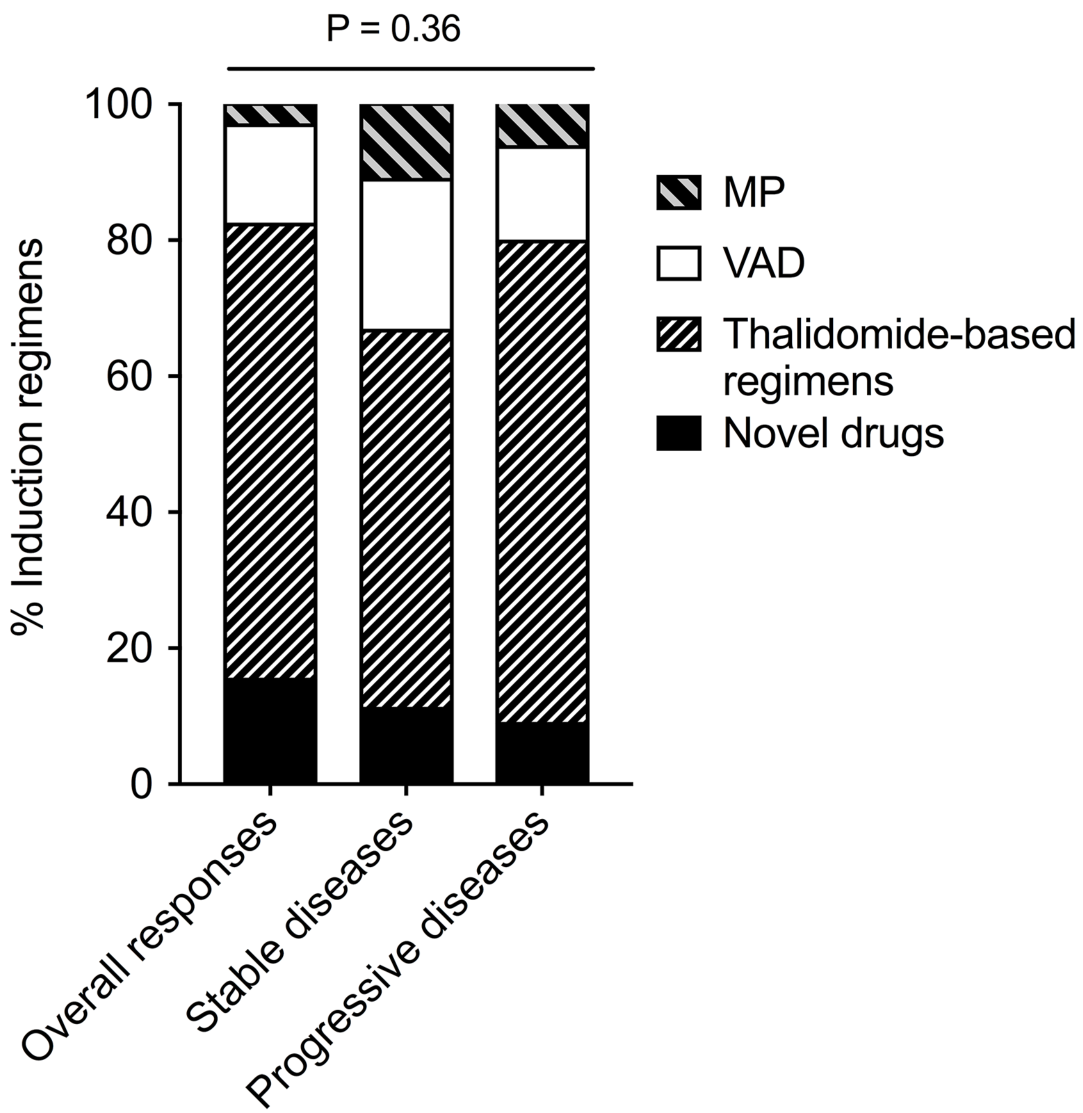

Supplementary Figure 4:The proportion of different induction therapies in patients with overall responses, stable diseases and progressive diseases. $p = 0.36$ by Pearson's $\chi 2$ test for the comparison between groups. *MP* melphalan with prednisone; *VAD* vincristine with adriamycin plus dexamethasone; Thalidomide-based regimens consisting of *TD* (thalidomide with dexamethasone) and *MPT* (melphalan with prednisone plus thalidomide); Novel drugs consisting of *LD* (lenalidomide with dexamethasone), *VD* (bortezomib with dexamethasone) and *PAD* (bortezomib with adriamycin plus dexamethasone).

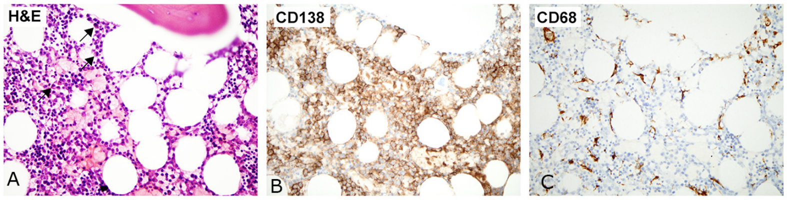

Supplementary Figure 5:Microscopic images of plasma cell rich areas in consecutive bone marrow sections from patient with MM. **(A)** H&E staining showed plasma cells (arrows indicated) distribution in morphology. **(B)** The bone marrow was in large areas packed with CD138 positive plasma cells (× 400 magnification). **(C)** The CD68 positive cells were seen in the bone marrow plasma cells rich areas (× 400 magnification).

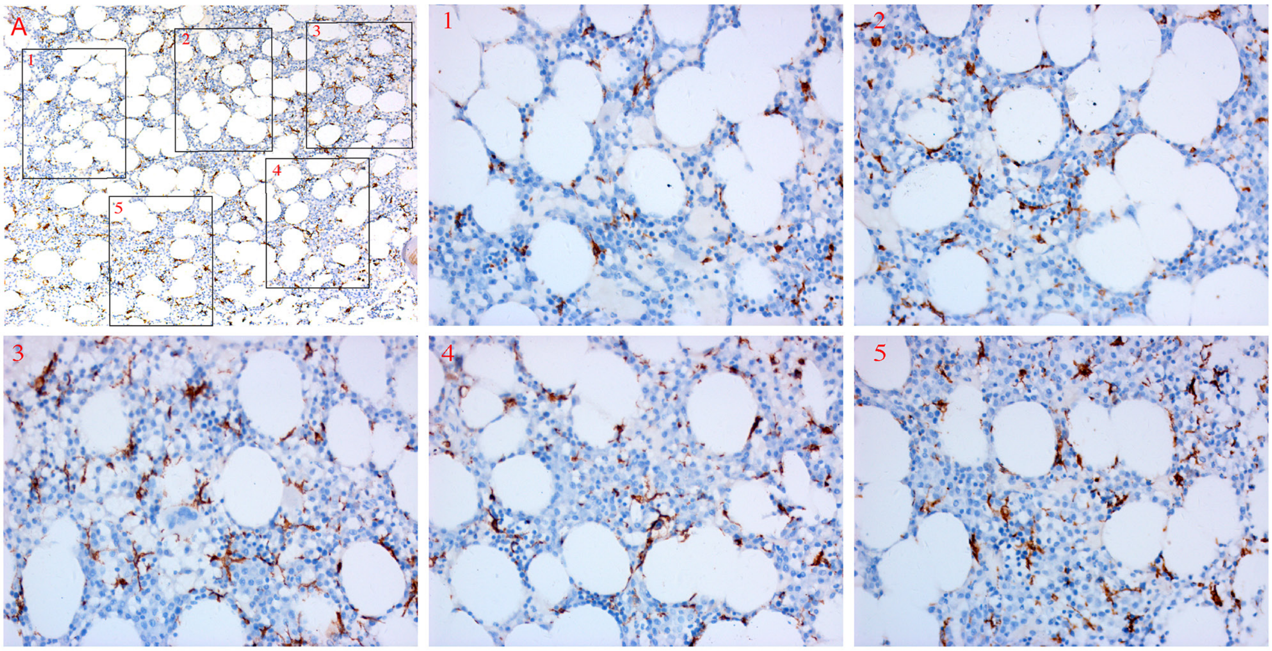

Supplementary Figure 6: Schematic images of CD68 positive TAMs hot spots chosen in bone marrow under microscope. **(A)** bone marrow IHC section evaluated at low magnification (× 100) and five representative hot spots (1-5) randomly chosen. The captured images of the five hot spots at 400 magnification shown as 1-5. The CD68 staining count of hot spot 1-5 were 36, 50, 67, 59 and 55, respectively.

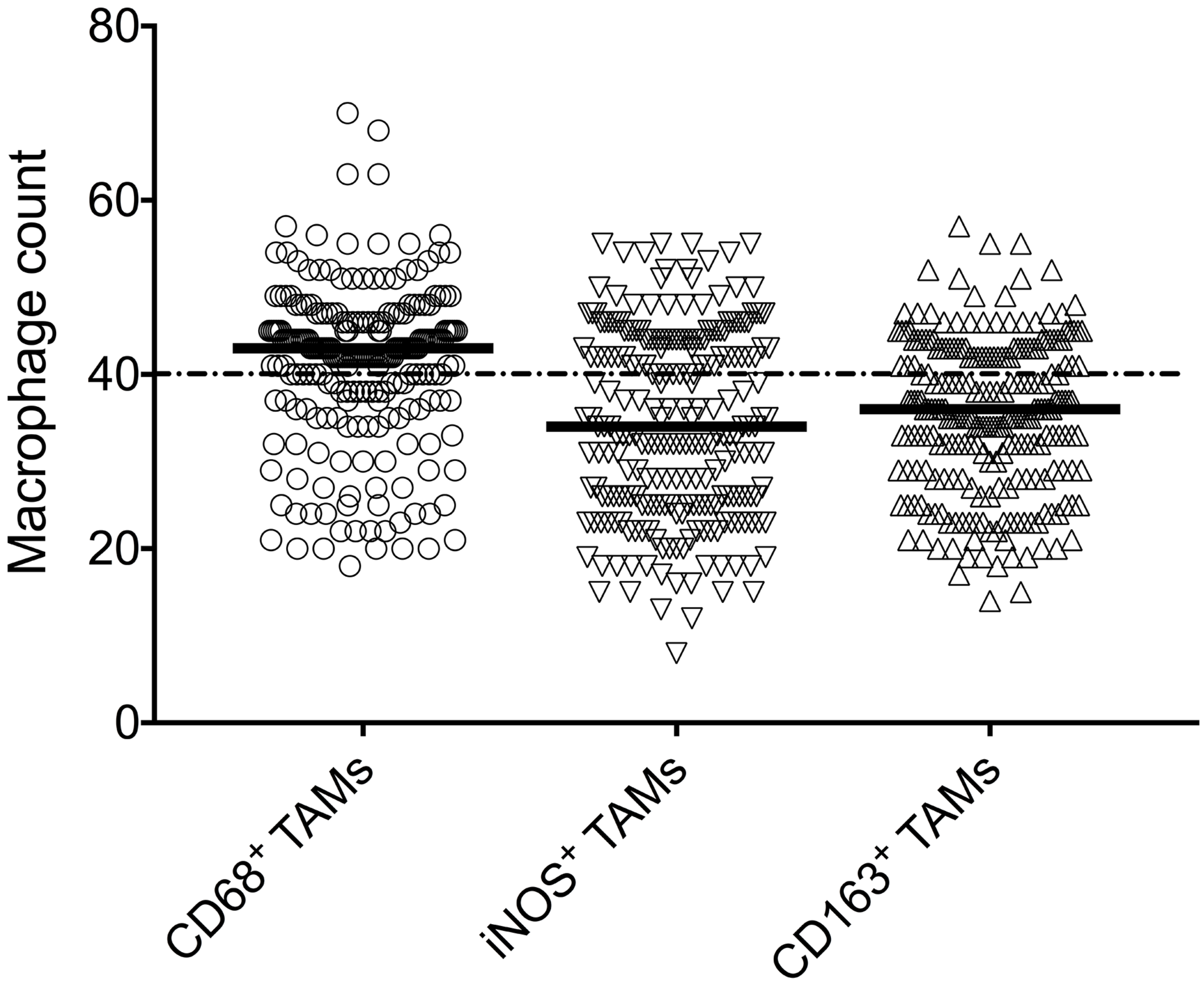

Supplementary Figure 7: The count distribution of three types of TAMs in 240 patients with myeloma (× 400). The median count (range) of CD68, iNOS and CD163 positive TAMs were 44 (17-70), 32 (8-54) and 36 (17-57), respectively.

Supplementary Table 1: Univariate and multivariate Cox regression analyses of potential prognostic factors for multiple myeloma.

Variable	Progression-free survival				Overall survival			
	Univariate analysis		Multivariate analysis		Univariate analysis		Multivariate analysis	
	HR (95 % CI)	*p*	HR (95 % CI)	*p*	HR (95 % CI)	*p*	HR (95 % CI)	*p*
Gender	1.00 (0.67-1.50)	1.00	0.93 (0.54-1.60)	0.78	1.13 (0.77-1.65)	0.54	0.83 (0.48-1.44)	0.51
Age	1.01 (0.99-1.03)	0.26	1.01 (0.99-1.03)	0.275	1.01 (0.99-1.03)	0.17	1.02 (1.00-1.04)	0.12
ISS		0.12		0.13		**0.002**		**0.001**
II vs. I	1.21 (0.57-3.27)	0.63	1.74 (0.92-3.28)	0.09	0.79 (0.35-1.79)	0.57	0.83 (0.36-1.90)	0.66
III vs. I	1.76 (0.95-1.81)	0.08	1.19 (0.55-2.56)	0.66	2.05 (1.12-3.76)	0.02	2.18 (1.18-4.04)	0.013
Creatinine	1.16 (0.77-1.77)	0.48	1.18 (0.75-1.85)	0.47	1.27 (0.86-1.84)	0.224	1.19 (0.79-1.79)	0.41
LDH	0.99 (0.65-1.53)	0.97	1.12 (0.60-2.09)	0.72	1.26 (0.86-1.85)	0.241	1.58 (0.87-2.85)	0.13
$CD68^+$ TAMs (high vs. low)	1.48 (0.97-2.26)	0.07	1.49 (0.97-2.29)	0.07	2.10 (1.44-3.07)	< **0.001**	2.18 (1.49-3.19)	<0.001

HR hazard ratio, *95 % CI* 95 % confidence interval, *ISS* International Staging System, *LDH* lactate dehydrogenase, *TAMs* tumor associated macrophages.

Supplementary Table 2: Correlations between ISS with clinicopathological characteristics.

Variable	ISS			
	**I (n = 35)**	**II (n = 45)**	**III (n = 160)**	***p***
Age				
Mean (years)[a]	59	63	63	0.13
Median	60.0	63.0	63	
Range	38-72	34-87	36-83	
Gender				0.25
Female	19 (54.3 %)	23 (51.1 %)	66 (41.3 %)	
Male	16 (45.7 %)	22 (48.9 %)	94 (58.7%)	
Creatinine (mg/dl)				0.90
≤ 2 mg/dl	21 (60 %)	29 (64.4 %)	102 (63.8 %)	
> 2 mg/dl	14 (40 %)	16 (35.6 %)	58 (36.2 %)	
LDH				0.47
Normal	20 (57.1 %)	26 (57.8 %)	105 (65.6 %)	
High	15 (42.9 %)	19 (42.2 %)	55 (34.4 %)	
Bone destruction				0.21
≤ 3 lesions	18 (51.4 %)	25 (55.6 %)	67 (41.9 %)	
> 3 lesions	17 (48.6 %)	20 (44.4 %)	93 (58.1 %)	

ISS International Staging System, *LDH* lactate dehydrogenase
[a] One way ANOVA; Chi-square test for all other analyses

Supplementary Table 3: Correlations between iNOS/CD163 signature with clinicopathological characteristics.

Variable	iNOS/CD163 signature				
	**I (n = 78)**	**II (n = 70)**	**II (n = 34)**	**II (n = 58)**	***p***
Age					
Mean (years)[a]	61.9	62.2	63.1	61.8	0.76
Median	62.5	63	64	59	
Range	34-87	36-81	36-83	44-83	
Gender					
Female	32 (41.0 %)	30 (42.9 %)	15 (44.1 %)	31 (53.4 %)	0.51
Male	46 (59.0 %)	40 (57.1 %)	19 (55.9 %)	27 (46.6 %)	
Creatinine (mg/dl)					
≤ 2 mg/dl	48 (61.5 %)	47 (67.1 %)	18 (52.9 %)	39 (67.2 %)	0.47
> 2 mg/dl	30 (38.5 %)	23 (32.9 %)	16 (47.1 %)	19 (33.8 %)	
LDH					
Normal	49 (62.8 %)	42 (60 %)	22 (64.7 %)	38 (65.5 %)	0.93
High	29 (37.2 %)	28 (40 %)	12 (35.3 %)	20 (34.5 %)	
Bone destruction					
≤ 3 lesions	35 (44.9 %)	36 (51.4 %)	15 (44.1 %)	24 (41.4 %)	0.74
> 3 lesions	43 (55.1 %)	34 (48.6 %)	19 (55.9 %)	34 (58.6 %)	
ISS					
I	12 (15.4 %)	11 (15.7 %)	6 (17.6%)	6 (10.3 %)	0.50
II	18 (23.1 %)	12 (17.1 %)	8 (23.5 %)	7 (12.1 %)	
III	48 (61.5 %)	47 (67.1 %)	20 (58.8 %)	45 (77.6 %)	

ISS International Staging System, *LDH* lactate dehydrogenase

[a] One way ANOVA; Chi-square test for all other analyses
